# Higher local Ebola incidence causes lower child vaccination rates

**DOI:** 10.1038/s41598-024-51633-3

**Published:** 2024-01-16

**Authors:** Upasak Das, David Fielding

**Affiliations:** Global Development Institute, University of Manchester, Manchester, M13 9PL UK

**Keywords:** Health policy, Health services

## Abstract

Ebola is a highly infectious and often fatal zoonotic disease endemic to West and Central Africa. Local outbreaks of the disease are common, but the largest recorded Ebola epidemic originated in Guinea in December 2013, spreading to Liberia, and Sierra Leone in the following year and lasting until April 2016. The epidemic presented a serious challenge to local healthcare systems and foreign aid agencies: it degraded services, caused the loss of healthcare professionals, disrupted the economy, and reduced trust in modern healthcare. This study aims to estimate the extent to which variation in one long-term measure of the quality of local healthcare (the child vaccination rate) is a consequence of local variation in the intensity of the epidemic. Applying a “difference-in-differences” model to household survey data from before and after the epidemic, we show that in 2018–2019, overall rates of vaccination for BCG, DPT, measles, and polio are lower in Guinean and Sierra Leonean districts that had a relatively high incidence of Ebola; statistical analysis indicates that this is a causal effect. The effects of the epidemic on access to healthcare have been local effects, at least in part.

## Introduction

Just as the mitigation of an epidemic depends on the capacity of local healthcare systems^1^, so the intensity of the epidemic can affect capacity, either through a contraction of the local economy and the resources available for healthcare, or through epidemic-related fatalities among healthcare professionals. The SARS-CoV-2 pandemic, for example, has strained healthcare systems across the world: even in resource-rich countries, there is a concern that collateral damage from the pandemic will persist for decades^2^. In West Africa, there is some evidence on the collateral consequences of epidemic disease during the outbreak of Ebola over 2014–2016. In the most severely affected countries (Guinea, Liberia, and Sierra Leone) there was an immediate reduction in the utilization of a wide range of healthcare services during the epidemic^3–8^. Researchers have expressed concern that the epidemic would also lead to a deterioration in health outcomes, either from reduced economic capacity^3,9,10^, Ebola-related fatalities among healthcare professionals^3,11^, lower trust in healthcare professionals^3,12–15^, or poor perinatal care^16–18^. One key element of child health is vaccination against measles and other infectious diseases, and there is case-study evidence that the Ebola epidemic has led to lower vaccination rates in specific locations^19,20^. One study conducted during the epidemic projected that if the observed reduction in measles vaccination rates observed during the epidemic persisted, then after 18 months, the consequent increase in measles incidence would cause an additional 2–16 K deaths^21^. Given recent local outbreaks of Ebola and the ongoing threat of future epidemics^22,23^, there is a risk of a vicious circle in which reduced healthcare capacity is both a cause and a consequence of higher disease prevalence.

A decline in vaccination rates following an epidemic could result from nationwide effects, e.g. nationwide reductions in healthcare capacity or trust, or from local effects, e.g. reductions in district-level healthcare capacity caused by a diminution of local financial resources or lower levels of trust that vary according to the extent of local healthcare failures. There was substantial variation in the local prevalence of Ebola in all three countries, partly (but not entirely) because of variation in population density^24^, and identifying the relative importance of local effects will be crucial to national efforts to mitigate any deterioration in healthcare capacity. To our knowledge, just one study^25^ has examined local variation in detail, using the difference-in-differences method with a sample of Sierra Leonean children. King et al. do not find any significant association of Ebola prevalence with vaccination rates, but the sample used is quite small.

The objective of this study is to estimate the size of local effects, i.e. the extent of within-country variation in vaccination rates caused by within-country variation in the intensity of the Ebola epidemic. More specifically, our objective is to measure the extent to which parents in areas with a high incidence of Ebola are less likely to vaccinate their children (which could be either because they are less willing to do this or because poor local capacity has made it more difficult). The estimates are produced by applying the difference-in-differences method to a larger sample of Sierra Leonean children and to samples of Guinean and Liberian children. As discussed in the methods section which follows, we examine the association of variation in local Ebola prevalence during the epidemic with variation in the number of fully vaccinated children and the number of vaccinations per child after the end of the epidemic. Our results section shows that (at least in Guinea and Sierra Leone) the local effects are substantial relative to the national trends. In other words, much of the effect of the epidemic on vaccination coverage has been through channels operating at a sub-national level. We conclude with a discussion of some of the implications of these results.

## Methods

### Data sources

This study employs data for children aged 13–35 months in the USAID Demographic Health Surveys (DHS); see https://dhsprogram.com^26^. Relevant data appear in survey rounds V (Guinea 2005, Liberia 2007, Sierra Leone 2008), VI (Guinea 2012, Liberia 2013, Sierra Leone 2013), and VII (Guinea 2018, Liberia 2019, Sierra Leone 2019); round IV data are also available for Guinea (1999). Children in rounds IV-VI were born and surveyed before the start of the epidemic; children in round VII were born during or after the end of the epidemic and surveyed at least 21 months afterwards. The surveys report whether a child has received the following WHO-recommended vaccinations: BCG, DPT (doses 1–3), measles (doses 1–2), and polio (doses 1–3). A fully vaccinated child therefore has nine vaccinations. Further information about the surveys appears in the Supplementary Materials.

Each survey uses a stratified sampling design: households are randomly selected from enumeration areas in each province of the country. These enumeration areas comprise one or two villages, or a suburb of a town. They are randomly selected from the towns and villages in a province, but the ratio of sample households to the total population of a province can vary from one province to another. For this reason, a sampling weight is attached to each enumeration area, and each child in a given enumeration area has the same sampling weight. Note, however, that the objective stated in the introduction relates to parental behaviour: our objective is to estimate the effect of the epidemic on decisions that parents make (or are forced to make). For this reason, we construct a sampling weight for each child that is equal to the enumeration area weight divided by the total reported number of children of that child’s mother. This weighting system ensures that our sample is representative of the population of mothers in each country, rather than the population of children. However, results using just the enumeration area weight (which are representative of the population of children) are very similar to those reported here. Overall, there were 9000 children in the Guinean sample, 6542 children in the Liberian sample, and 9255 children in the Sierra Leonean sample, but some observations were excluded from the analysis because of missing covariate values (455 in Guinea, 539 in Liberia, and 345 in Sierra Leone).

### Data analysis

**Figure 1:**
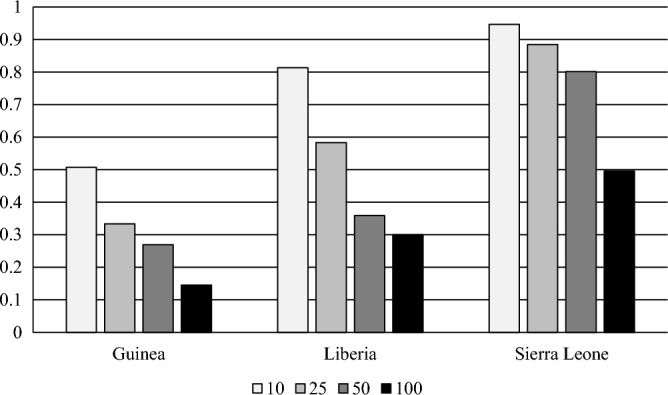
The fraction of children in each national sample inhabiting high-incidence districts. The figure shows fractions for four different definitions of high incidence at the district level: > 10 per 100 K, > 25 per 100 K, > 50 per 100 K, and > 100 per 100 K. The samples are Guinean children aged 12–35 months in DHS rounds IV–VII, Liberian children aged 13–35 months in DHS rounds V–VII, and Sierra Leone children aged 12–35 months in rounds V-VII.

The data analysis is based on a comparison of vaccination rates before and after the epidemic in districts that had a relatively high incidence of Ebola with those that had a relatively low incidence. (“District” indicates a major administrative division of a country, i.e. one of the 34 Guinean prefectures, 15 Liberian counties, or 14 Sierra Leone districts.) The definition of high incidence varies across the three countries, for the reasons illustrated in Fig. 1. Using a threshold of 10 per 100 K in Guinea means that about 50% of children in the sample live in high-incidence districts, giving some robustness to tests of the significance of differences between high-incidence and low-incidence districts. However, using this threshold in Sierra Leone means that approximately 95% of children live in high-incidence districts, and results based on this threshold are unlikely to be robust. In the results below, the threshold used in each country places over 30% and under 70% of children in high-incidence districts. For Guinea, there are two alternative thresholds: 10 per 100 K and 25 per 100 K. For Liberia, the alternative thresholds are 25 per 100 K and 50 per 100 K. For Sierra Leone, the results are based on a threshold of 100 per 100 K. Results using other thresholds appear in the Supplementary Materials. The district-specific incidence figures are taken from Fig. 1 of Dahl et al.^27^, which is based on World Health Organization data for the confirmed, probable and suspected number of cases per 100 K population. See WHO Ebola Response Team (2014) for more details regarding the derivation of the data^28^. The association of vaccination rates with Ebola incidence is measured using a “difference-in-differences” model^29^. The post-epidemic difference in vaccination rates between high-incidence and low-incidence districts is compared with the pre-epidemic difference. The change in the difference, conditional on observable child characteristics, is interpreted as the effect of the epidemic.

The difference-in-differences model allows for the possibility that changes in mean child characteristics varied systematically between high-incidence and low-incidence districts, and that these characteristics were themselves associated with vaccination rates. The vaccination status of each child in each round of the DHS is modelled as a function of (i) the observed characteristics of the child and its household, (ii) whether the child was surveyed in round VII, (iii) whether the child was living in a district that had (or would later have) a high Ebola incidence, and (iv) the interaction of (ii) and (iii). The coefficient on this interaction term measures the difference in differences, capturing the effect of living in a high-incidence district after the epidemic. This is a conservative estimate of the effect of the epidemic, because it excludes any indirect effect through an association of the characteristics in (i) with Ebola incidence.

In each country, the difference-in-differences model is fitted to a pooled sample of children across all rounds of the survey (i.e. rounds IV-VII in Guinea and rounds V-VII in the other two countries). There are two versions of the model, the first of which estimates the effect of the epidemic on the probability that the *it*h child is fully vaccinated using the following Probit function:

1 $${\text{P}}\left( {i\,\,{\text{vaccinated}}} \right) = \Phi \left( {\sum\nolimits_{k} {\beta_{k} \cdot x_{ki} + \varphi_{1} \cdot seven_{i} + \varphi_{2} \cdot Ebola_{i} + \varphi_{3} \cdot seven_{i} \cdot Ebola_{i} } } \right)$$

P(*i* vaccinated) is the probability that the *it*h child is fully vaccinated and Φ(.) is the cumulative normal density function. *Ebola*_*i*_ = 1 if child *i* was living in a high-incidence district, otherwise *Ebola*_*i*_ = 0; *seven*_*i*_ = 1 if the child was surveyed in round VII of the DHS, otherwise *seven*_*i*_ = 0. Each of the variables *x*_*ki*_ (*k* = 1,…, *K*) is a characteristic of the child or its household. These characteristics are discussed in the Supplementary Materials; they include the child’s age, sex and birth order, the mother’s age, education level and religion, the household wealth level, the age and sex of its head, and the local population density and rainfall level. The *β* and *φ* terms are parameters to be estimated. Equation (1) is fitted to the data using the child-specific sampling weights described above.

The size of the effect of living in an Ebola-affected district after the epidemic on the probability of being fully vaccinated depends on the value of $\varphi_{3} ,$ but the effect is not uniform across all districts, because Φ(.) is a non-linear function^30^. The average effect is computed by applying the following formula to the sample of round-VII children:

2 $${{\sum\nolimits_{i = 1}^{i = M} {\left( {\Phi \left( {\sum\nolimits_{k} {\hat{\beta }_{k} \cdot x_{ki} + \hat{\varphi }_{1} + \hat{\varphi }_{2} \cdot Ebola_{i} + \hat{\varphi }_{3} } } \right) - \,\Phi \left( {\sum\nolimits_{k} {\hat{\beta }_{k} \cdot x_{ki} + \hat{\varphi }_{1} + \hat{\varphi }_{2} \cdot Ebola_{i} } } \right)} \right)} } \mathord{\left/ {\vphantom {{\sum\nolimits_{i = 1}^{i = M} {\left( {\Phi \left( {\sum\nolimits_{k} {\hat{\beta }_{k} \cdot x_{ki} + \hat{\varphi }_{1} + \hat{\varphi }_{2} \cdot Ebola_{i} + \hat{\varphi }_{3} } } \right) - \,\Phi \left( {\sum\nolimits_{k} {\hat{\beta }_{k} \cdot x_{ki} + \hat{\varphi }_{1} + \hat{\varphi }_{2} \cdot Ebola_{i} } } \right)} \right)} } M}} \right. \kern-0pt} M}$$

Here, *M* denotes the number of children in round VII and hats denote parameter estimates. Values of $\hat{\varphi }_{3}$ appear in the Supplementary Materials.

The second version of the model estimates the effect of the epidemic model on the number of vaccinations received by child *i*, expressed as a fraction of the total possible number (nine). A fractional Probit model is fitted to the data^31^:

3 $${\text{E}}\left[ {vaccinations_{i} } \right] = \Phi \left( {\sum\nolimits_{k} {\delta_{k} \cdot x_{ki} + \eta_{1} \cdot seven_{i} + \eta_{2} \cdot Ebola_{j} + \eta_{3} \cdot seven_{i} \cdot Ebola_{j} } } \right)$$

E[*vaccinations*_*i*_] is the expected fraction for child *i*, and the *δ* and *η* terms are parameters to be estimated. Again, the estimated effect of living in an Ebola-affected district is not uniform across districts, so the average effect is computed by applying the following formula to the sample of round-VII children:

4 $${{\sum\nolimits_{i = 1}^{i = M} {\left( {\Phi \left( {\sum\nolimits_{k} {\hat{\delta }_{k} \cdot x_{ki} + \hat{\eta }_{1} + \hat{\eta }_{2} \cdot Ebola_{i} + \hat{\eta }_{3} } } \right) - \,\Phi \left( {\sum\nolimits_{k} {\hat{\delta }_{k} \cdot x_{ki} + \hat{\eta }_{1} + \hat{\eta }_{2} \cdot Ebola_{i} } } \right)} \right)} } \mathord{\left/ {\vphantom {{\sum\nolimits_{i = 1}^{i = M} {\left( {\Phi \left( {\sum\nolimits_{k} {\hat{\delta }_{k} \cdot x_{ki} + \hat{\eta }_{1} + \hat{\eta }_{2} \cdot Ebola_{i} + \hat{\eta }_{3} } } \right) - \,\Phi \left( {\sum\nolimits_{k} {\hat{\delta }_{k} \cdot x_{ki} + \hat{\eta }_{1} + \hat{\eta }_{2} \cdot Ebola_{i} } } \right)} \right)} } M}} \right. \kern-0pt} M}$$

Values of $\hat{\eta }_{3}$ appear in the Supplementary Materials.

It will also be necessary to construct confidence intervals around the estimates in Eqs. (2) and (4). In both cases, these confidence intervals are based on standard errors of the parameter estimates that allow for clustering at the district level.

Performing precise ex ante power calculations in this type of model is problematic, because such calculations depend on assumptions about the values of all of the *β*_*k*_ and *δ *_*k*_ parameters, and for some child characteristics, evidence on these values is very limited. Nevertheless, previous studies of factors affecting the probability of vaccination using other DHS samples with similar samples sizes and estimation methods (but focussing on factors other than exposure to previous epidemics) have reported significant effects (p < 0.05), even when the estimated effect size is a difference in vaccination rates of less than five percentage points^32,33^. It is therefore reasonable to expect that any effect of five percentage points or more in the population could be identified by our method.

Implicit in our estimation method is the assumption that, in the absence of the treatment (high Ebola incidence versus low Ebola incidence), the difference in vaccination rates between the treated and untreated locations (conditional on the covariates *x*_*ki*_) would have remained constant. In other words, it is assumed that the trend over time the vaccination measure would have been the same in the treated and untreated locations. One way to examine the plausibility of this assumption is to measure the trends in the treated and untreated locations over the sample periods preceding the epidemic. If the trends are parallel, then the assumption is more credible. The Supplementary Materials include an exercise of this kind. In one case (the probability of being fully vaccinated in Sierra Leone), the hypothesis of parallel trends can be rejected at the five percent level. However, in all other cases (including the number of vaccinations in Sierra Leone), the hypothesis cannot be rejected, suggesting that our results are robust overall.

## Results

**Figure 2:**
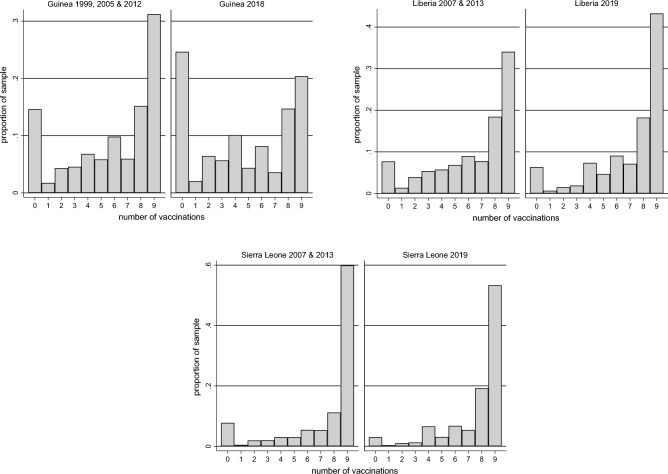
Distribution of the number of vaccinations per child. In each country, the left-hand panels show the distribution for children aged 13–35 months in DHS rounds IV (Guinea only), V and VI. The right-hand panels show the distribution in DHS round VII. The vaccinations are for BCG, DPT (doses 1–3), measles (doses 1–2), and polio (doses 1–3).

Before presenting the difference-in-differences results, we observe that the data show some variation in both the level of and change in overall vaccination rates across the three countries. This is illustrated in Fig. 2, which depicts the distribution of the number of vaccinations per child in each country before the epidemic (rounds IV-VI) and afterwards (round VII). In Guinea, there was a fall in the mean number of vaccinations per child from 5.8 across 1999, 2005 and 2012 to 4.7 in 2018. In Liberia, there was a rise in the mean from 6.4 across 2007 and 2013 to 7.0 in 2019. In Sierra Leone, there was a rise in the mean from 7.4 across 2007 and 2013 to 7.6 in 2019. The Guinean data indicate a more persistent decline in vaccination rates than suggested by earlier WHO estimates^30^. Using a difference-in-differences model, we can estimate the extent to which local variation around these means is associated with local variation in Ebola incidence.

**Figure 3:**
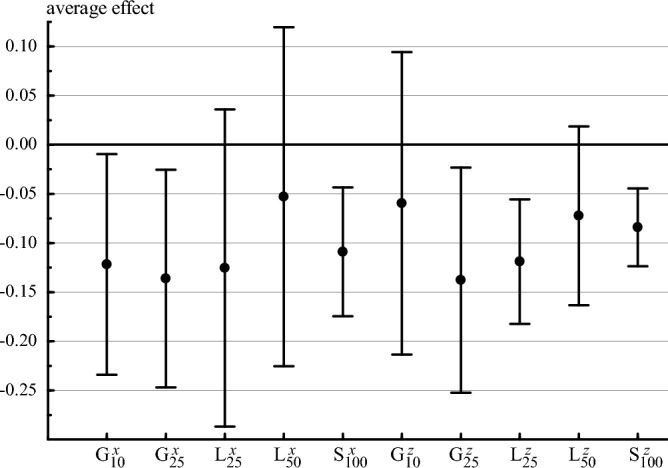
Average effects of living in a high-incidence district. Each dot represents a different estimate of the average effect of inhabiting a high-incidence district after the epidemic; bars represent the corresponding 95 percent confidence intervals. G indicates an estimate for Guinea; L indicates an estimate for Liberia; S indicates an estimate for Sierra Leone. Superscript *x* indicates an average effect on the probability of being fully vaccinated; superscript *z* indicates an average effect on the fraction of vaccinations received. Subscript 10 indicates a threshold incidence of 10 per 100 K to define high incidence; subscript 25 indicates a threshold of 25 per 100 K; subscript 50 indicates a threshold of 50 per 100 K; subscript 100 indicates a threshold of 100 per 100 K. The Guinean sample comprises children aged 13–35 months in DHS rounds IV–VII (N = 8555); the Liberian and Sierra Leone samples comprise children aged 13–35 months in DHS rounds V–VII (N = 6013 and N = 8910 respectively).

The results are summarized in Fig. 3, which includes estimates of a number of different effects. First, the figure shows estimates of average effect of living in a high-incidence district on the probability of being fully vaccinated, i.e. having all the vaccinations listed above; these estimates employ the formula in Eq. (2). Second, using Eq. (4), there are estimates of the average effect on the number of vaccinations received, expressed as a fraction of the recommended number (i.e. nine). Using either a 10 per 100 K or a 25 per 100 K threshold, living in a high-incidence district in Guinea is estimated to reduce the probability of being fully vaccinated by approximately 13 percentage points, on average; both effects are statistically significant (*p* < 0.05). Using a threshold of 10 per 100 K, there is no significant effect on the number of vaccinations, but using a threshold of 25 per 100 K, there is a significant effect (*p* < 0.05): living in a high-incidence district is estimated to reduce the vaccination fraction by approximately 14 percentage points, i.e. approximately 1.2 vaccinations. This is a large effect, considering that the nationwide post-Ebola mean is 4.7 vaccinations per child.

Figure 3 also shows that living in a high-incidence district in Sierra Leone is estimated to reduce the probability of being fully vaccinated by approximately 11 percentage points and the vaccination fraction by approximately 8 percentage points, i.e. approximately 0.7 vaccinations. Both effects are statistically significant (*p* < 0.01), although, given the parallel trends analysis noted above, the second estimate may be more robust than the first. The estimated effects in Liberia are also negative but are mostly insignificantly different from zero.

## Discussion

Previous studies indicate that there was a large fall in national child vaccination rates during the Ebola epidemic. In particular, Sun et al. report that during the epidemic, the proportion of Sierra Leonean children fully vaccinated against measles fell by 26 percentage points, with a partial recovery in vaccination coverage over 2015^19^. Similarly, Wesseh et al. report a fall in Liberian vaccination coverage of 37 percentage points during the epidemic, followed by a partial recovery over 2015^20^. These data are broadly consistent with those in Masresha et al., which indicate similar patterns in national vaccination rates across Guinea, Liberia and Sierra Leone^34^. Our data indicate that by 2018–2019, national vaccination rates in Liberia and Sierra Leone were similar to their pre-epidemic levels: see Fig. 2. However, these results tell us nothing about the local variation in vaccination coverage within each country. Wesseh et al. do compare changes in Liberian vaccination rates in areas of high Ebola incidence with those in areas of low incidence, finding (somewhat surprisingly) that recovery towards pre-epidemic vaccination rates was faster in high-incidence areas. However, this study does not control for other factors, such as urbanisation, that may have influenced both the severity of the epidemic and the ability of an area to reconstruct its healthcare system. Our results indicate that when such factors are taken into account, there is a large, negative and statistically significant association between the local severity of the epidemic and child vaccination rates in Guinea and Sierra Leone 2–3 years after the end of the epidemic. Controlling for other factors, the proportion of fully vaccinated Guinean children in high-incidence districts is estimated to have been 13 percentage points lower than the proportion in low-incidence districts; the corresponding figure in Sierra Leone is 11 percentage points. (The estimates for Liberia are similar in magnitude but rather less precise and therefore statistically insignificant.) While these differences are somewhat smaller than the national decline in vaccination rates during the epidemic, they still indicate substantial regional variation. In the 2–3 years following the end of the epidemic, its effect on healthcare services has not been uniform across each country, and locations with a relatively high incidence of infectious disease experience a much larger decline in the use of services. To the extent that these services protect a local community from future infection, there is likely to be increasing within-country inequality in health outcomes, unless the country’s healthcare policy takes local heterogeneity into account.

Evidence from previous research indicates the successful deployment of a large number of healthcare workers after the epidemic, but the effectiveness of the deployment has been inhibited by poor co-ordination between local and national managers^35^. The local variation in vaccination rates that we have uncovered underscores the importance of co-ordination, so that new healthcare workers are deployed to the places where they are most needed. However, the main limitation of our study is that we have not yet identified the extent to which the causal channels behind the local variation relate to the “supply side” (higher local Ebola incidence having led to more degradation of local health services) or to the “demand side” (parents in high-incidence areas being more distrustful of health professionals). For this, a structural model of vaccine supply and demand will be required. The development of district-specific interventions will depend on a better understanding of the relative importance of supply-side and demand-side effects. If supply-side effects dominate, then there is a case for regionally targeted expenditure to rebuild local healthcare systems, for example by prioritising the deployment of newly trained nurses to specific locations. If demand-side effects dominate, then there is a case for regional targeting of policies to address vaccine hesitancy among parents, for example through new forms of communication by public healthcare professionals.

We note as a caveat that the covariates *x*_*ki*_ included in the model might not fully capture all factors of the incidental characteristics associated with both Ebola incidence and vaccination rates. For example, for a given population density, the quality of an area’s transportation infrastructure may be associated with both ease of access to healthcare facilities and the speed at which infections are transmitted. Further information on such factors would improve the reliability of the model.

### Supplementary Information

Supplementary Information.

## Data Availability

Data are available for download from the DHS; see the Supplementary Materials for more details.
