# Supplementary material for: Higher local Ebola incidence causes lower child vaccination rates
Source: Sci Rep. 2024 Jan 16;14:1382. doi: 10.1038/s41598-024-51633-3 (PMC10791637; doi:10.1038/s41598-024-51633-3)
Supplement: Supplementary file 1 — Supplementary Information. [file 41598_2024_51633_MOESM1_ESM.docx]

**Supplementary Materials**

**S1. Data**

Table S1 lists the covariates *k* used in the statistical model outlined in Figure 3 of the main text. These are child characteristics taken from the following USAID Demographic and Health Surveys. All of these variables are included in our statistical model. Note, however, that ethnicity is not reported in the Liberian DHS and so does not appear in the model for Liberia.

*Guinea* 1999: https://dhsprogram.com/methodology/survey/survey-display-154.cfm

2005: https://dhsprogram.com/methodology/survey/survey-display-249.cfm

2012: https://dhsprogram.com/methodology/survey/survey-display-391.cfm

2018: https://dhsprogram.com/methodology/survey/survey-display-539.cfm

*Liberia* 2007: https://dhsprogram.com/methodology/survey/survey-display-271.cfm

2013: https://dhsprogram.com/methodology/survey/survey-display-435.cfm

2019: https://dhsprogram.com/methodology/survey/survey-display-537.cfm

*Sierra Leone* 2008: https://dhsprogram.com/methodology/survey/survey-display-324.cfm

2013: https://dhsprogram.com/methodology/survey/survey-display-450.cfm

2019: https://dhsprogram.com/methodology/survey/survey-display-545.cfm

Tables S2-S4 include the mean values of each covariate in each individual survey. Table S5 shows the total number of observations in each prefecture or district in the final estimation sample, after children for whom some covariate data are missing have been excluded. Table S6 shows the district-specific estimated ranges of Ebola incidence in Dahl et al. (reference 27 in the main text).

**S2. Vaccination rates in areas of low Ebola incidence and in areas of high Ebola incidence**

Table S7 shows (i) the proportion of children fully vaccinated and (ii) the mean number of vaccinations per child in prefectures / districts with high Ebola incidence and in prefectures / districts with low Ebola incidence. Data are shown separately for each country, using the same country-specific definitions of high incidence and low incidence as in Figure 3 of the main text. The means and proportions are calculated across all rounds of the DHS survey. It can be seen that in Guinea and Liberia, mean vaccination rates are higher in the high-incidence districts. This reflects that fact that certain characteristics (in particular population density) are associated with both a high incidence of infectious disease and better access to healthcare services. It is important to control for such characteristics when estimating the effect of Ebola incidence on vaccination rates.

**S3. Exploring the assumption of parallel trends**

The difference-in-differences method assumes that, in the absence of the treatment, the trend over time the vaccination rate (conditional on the covariates) would have been the same in the treated and untreated locations (i.e. the high-incidence and low-incidence districts). One way of exploring the plausibility of this assumption is to include in the statistical model not only the interaction *seven_i_* ⋅ *Ebola_i_*, but also the interaction of *Ebola_i_* with indicator variables for other survey years. In the case of Guinea, this involves including two extra interaction terms (for 2005 and 2012); the in case of Liberia and Sierra Leone, it involves including one extra interaction term (for 2013). If the parallel trends assumption is correct, then the expected estimate of the effect of each extra interaction term on the probability of being fully vaccinated (or on the number of vaccinations per child) is equal to zero. Figures S1-S3 present estimates of the effects, along with the corresponding 95 percent confidence intervals.

Fig. S1 shows the estimates for Guinea. Estimates are shown for the models using thresholds of 10 per 100K and 25 per 100K, i.e. those used in Figure 3 of the main text. The charts on the left-hand side of Figure S1 show that with a 10 per 100K threshold, the estimated effects of the additional interaction terms (for 2005 and 2012) are very close to zero. For both outcomes – the probability of vaccination and the number of vaccinations (expressed as a fraction of the maximum possible, i.e. 9) – the additional effects are individually and jointly insignificant at the 5 percent level. In other words, the difference in vaccination rates between those districts that would later have a high incidence of Ebola and those that would not was not significantly larger in 2005 (or in 2012) than in 1999. Figure S1 also shows the estimated effect of the 2018 interaction, but note that this estimate has a different interpretation to the one in Figure 3 of the main text. Figure 3 shows the difference between vaccination rates in high-incidence districts in 2018 and those in high-incidence districts in all three previous survey years; Figure S1 shows the difference between vaccination rates in high-incidence districts in 2018 and those in high-incidence districts in 1999. The second comparison is based on a smaller number children in the reference sample.

The right-hand side of Figure S1 shows estimates with a 25 per 100K threshold. The estimated effects of the additional interaction terms are again jointly and individually insignificantly different from zero at the 5 percent level. However, the estimated effect for 2012 is significantly different from zero at the 10 percent level. For this reason, the Figure 3 results for the 10 per 100K threshold may be more reliable.

Fig. S2 shows the corresponding results for Liberia, where none the additional interaction effects (for 2013) is significantly different from zero. Figure S3 show the corresponding results for Sierra Leone. Here, the estimate of the additional interaction effect on the probability of being fully vaccinated is significantly different from zero. However, the estimate of the additional interaction effect on the vaccination fraction is precisely equal to zero. For this reason, the Figure 3 results for the vaccination fraction may be more reliable.

**S4. Results using alternative thresholds to define a high incidence of Ebola**

Table S8 shows the full set of estimates of and $\hat{\eta}_{3}$ (see the notes to Figure 3 of the main text). The table also shows the corresponding standard errors and average effects, computed using the method described in Figure 3.

| Table S1. Definitions of covariates used in the statistical model | |
| --- | --- |
|  |  |
| Variable | Definition |
|  |  |
| Age | Age of the child on the day of the interview (in years, reported to the nearest month) |
| Girl | = 1 if the child is female; 0 otherwise |
| Birth order of the child | Birth order of the child (i.e. 1 for the first child, 2 for the second child, and so on) |
| Mothers’ age | Age of the child’s mother of the child in years |
| Mothers’ education | Educational level of the child’s mother (0: no education; 1: primary; 2: secondary; 3: higher secondary or above) |
| Muslim (for Guinea and Sierra Leone) | = 1 if the religion that the child’s household practices is Islam religion; 0 otherwise |
| Christian (for Liberia) | = 1 if the religion that the child’s household practices is Christianity; 0 otherwise |
| No improved toilet | = 0 if the child’s household has the following type of the toilet facility: flush to piped sewer system / septic tank / pit latrine / somewhere else / flush to somewhere which the respondent does not know / ventilated improved pit (vip) / biogas latrine / pit latrine with slab; = 1 for the following types of facility: pit latrine without slab / open pit / twin pit / composting toilet / dry toilet / no facility / uses open space or field |
| Earth floor | = 1 if the main material of the floor of the child’s household is made from mud, clay or earth; = 0 if the main material is the following: raw wood planks, palm, bamboo, brick, stone, vinyl, asphalt, ceramic tiles, cement, carpet, polished stone / marble / granite |
| *Wealth quantile* |  |
| Quantile 1 (poorest) | =1 if the wealth index group as categorized by the DHS is 1; 0 otherwise |
| Quantile 2 | =1 if the wealth index group as categorized by the DHS is 2; 0 otherwise |
| Quantile 3 | =1 if the wealth index group as categorized by the DHS is 3; 0 otherwise |
| Quantile 4 | =1 if the wealth index group as categorized by the DHS is 4; 0 otherwise |
| Quantile 5 (richest) | =1 if the wealth index group as categorized by the DHS is 5; 0 otherwise |
|  | Construction of the index is described in S.O Rutstein & K. Johnson, The DHS wealth index. DHS Comparative Reports 6, Calverton, Maryland: ORC Macro (2004). See https://dhsprogram.com/pubs/pdf/CR6/CR6.pdf |
|  |  |
| Female household head | = 1 if head of the child’s household is female; 0 otherwise |
| Age of household head | Age of the head of the child’s household head in years |
| Urban | = 1 if the place of residence of the child’s household is urban; 0 otherwise |
| Population density | The log of the population density index of the child’s cluster, as described in A.J. Florczyk, C. Corbane, D. Ehrlich, S. Freire, T. Kemper, L. Maffenini, M. Melchiorri, M. Pesaresi, P. Politis, M. Schiavina, F. Sabo & L. Zanchetta, GHSL Data Package 2019. EUR 29788 EN, Publications Office of the European Union: Luxembourg (2019). |
| Rainfall | Mean annual rainfall in the child’s cluster in millimeters |
|  |  |
| Table S1 Definitions of covariates used in the statistical model (continued) | |
|  |  |
| Variable | Definition |
|  |  |
| *Ethnicity (for Guinea)* |  |
| Soussou | = 1 if the ethnicity of the child’s household is Soussou; 0 otherwise |
| Peulh | = 1 if the ethnicity of the child’s household is Peulh; 0 otherwise |
| Malink | = 1 if the ethnicity of the child’s household is Malink; 0 otherwise |
| Kissi | = 1 if the ethnicity of the child’s household is Kissi; 0 otherwise |
| Toma | = 1 if the ethnicity of the child’s household is Toma; 0 otherwise |
| Guerz | = 1 if the ethnicity of the child’s household is Guerz; 0 otherwise |
| Others | = 1 if the ethnicity of the child’s household is none of the above; 0 otherwise |
|  |  |
| *Ethnicity (for Sierra Leone)* | |
| Creole | = 1 if the ethnicity of the child’s household is Creole; 0 otherwise |
| Fullah | = 1 if the ethnicity of the child’s household is Fullah; 0 otherwise |
| Kono | = 1 if the ethnicity of the child’s household is Kono; 0 otherwise |
| Limba | = 1 if the ethnicity of the child’s household is Limba; 0 otherwise |
| Loko | = 1 if the ethnicity of the child’s household is Loko; 0 otherwise |
| Mandingo | = 1 if the ethnicity of the child’s household is Mandingo; 0 otherwise |
| Mende | = 1 if the ethnicity of the child’s household is Mende; 0 otherwise |
| Sherbro | = 1 if the ethnicity of the child’s household is Sherbro; 0 otherwise |
| Temne | = 1 if the ethnicity of the child’s household is Temne; 0 otherwise |
| Korankoh | = 1 if the ethnicity of the child’s household is Korankoh; 0 otherwise |
| Others | = 1 if the ethnicity of the child’s household is none of the above; 0 otherwise |
|  |  |

| Table S2. Descriptive statistics for Guinea | | | | | | | | | | | | | |
| --- | --- | --- | --- | --- | --- | --- | --- | --- | --- | --- | --- | --- | --- |
|  |  | | | | | | | | | |  | | |
|  | **Pre-Ebola** | | | **Pre-Ebola** | | | **Pre-Ebola** | | | | **Post-Ebola** | | |
|  | 1999 | | | 2005 | | | 2012 | | | | 2018 | | |
|  | *Observations* | *Mean* | | *Observations* | | *Mean* | *Observations* | | *Mean* | | *Observations* | | *Mean* |
|  |  |  | |  | |  |  | |  | |  | |  |
| Fully immunized | 1,806 | 0.256 | | 2,121 | | 0.326 | 2,443 | | 0.334 | | 2,630 | | 0.203 |
| Age | 1,806 | 1.862 | | 2,121 | | 1.916 | 2,443 | | 1.881 | | 2,630 | | 1.848 |
| Girl | 1,806 | 0.485 | | 2,121 | | 0.482 | 2,443 | | 0.483 | | 2,630 | | 0.481 |
| Birth order of the child | 1,806 | 3.970 | | 2,121 | | 3.983 | 2,443 | | 3.689 | | 2,630 | | 3.500 |
| Mother’s age | 1,806 | 28.468 | | 2,121 | | 29.331 | 2,443 | | 28.198 | | 2,630 | | 28.670 |
| Mother’s education | 1,806 | 0.194 | | 2,121 | | 0.165 | 2,443 | | 0.336 | | 2,630 | | 0.420 |
| Muslim | 1,794 | 0.844 | | 2,121 | | 0.886 | 2,436 | | 0.899 | | 2,630 | | 0.899 |
| Improved toilet | 1,773 | 0.834 | | 2,045 | | 0.305 | 2,409 | | 0.592 | | 2,601 | | 0.519 |
| Earth floor | 1,791 | 0.582 | | 2,047 | | 0.615 | 2,410 | | 0.481 | | 2,598 | | 0.408 |
|  |  |  | |  | |  |  | |  | |  | |  |
| *Wealth quantile* |  |  | |  | |  |  | |  | |  | |  |
| Quantile 1 (poorest) | 1,767 | 0.378 | | 2,121 | | 0.229 | 2,443 | | 0.230 | | 2,630 | | 0.235 |
| Quantile 2 | 1,767 | 0.134 | | 2,121 | | 0.235 | 2,443 | | 0.203 | | 2,630 | | 0.220 |
| Quantile 3 | 1,767 | 0.311 | | 2,121 | | 0.216 | 2,443 | | 0.212 | | 2,630 | | 0.183 |
| Quantile 4 | 1,767 | 0.008 | | 2,121 | | 0.195 | 2,443 | | 0.227 | | 2,630 | | 0.199 |
| Quantile 5 (richest) | 1,767 | 0.169 | | 2,121 | | 0.124 | 2,443 | | 0.127 | | 2,630 | | 0.162 |
|  |  |  | |  | |  |  | |  | |  | |  |
| Female household head | 1,806 | 0.087 | | 2,121 | | 0.104 | 2,443 | | 0.118 | | 2,630 | | 0.128 |
| Age of household head | 1,804 | 45.895 | | 2,120 | | 46.566 | 2,441 | | 46.601 | | 2,630 | | 45.636 |
| Urban | 1,806 | 0.251 | | 2,121 | | 0.209 | 2,443 | | 0.290 | | 2,630 | | 0.294 |
| Population density | 1,806 | -5.813 | | 2,121 | | -6.995 | 2,443 | | -5.304 | | 2,630 | | -5.246 |
| Rainfall | 1,806 | 2068.9 | | 2,121 | | 1843.9 | 2,443 | | 2149.4 | | 2,630 | | 2047.5 |
|  |  |  | |  | |  |  | |  | |  | |  |
| *Ethnicity* |  |  | |  | |  |  | |  | |  | |  |
| Soussou | 1,806 | 0.168 | | 2,121 | | 0.183 | 2,443 | | 0.153 | | 2,630 | | 0.179 |
| Peulh | 1,806 | 0.331 | | 2,121 | | 0.384 | 2,443 | | 0.385 | | 2,630 | | 0.393 |
| Malink | 1,806 | 0.324 | | 2,121 | | 0.292 | 2,443 | | 0.333 | | 2,630 | | 0.314 |
| Kissi | 1,806 | 0.052 | | 2,121 | | 0.047 | 2,443 | | 0.056 | | 2,630 | | 0.049 |
| Toma | 1,806 | 0.023 | | 2,121 | | 0.036 | 2,443 | | 0.009 | | 2,630 | | 0.010 |
| Guerz | 1,806 | 0.087 | | 2,121 | | 0.050 | 2,443 | | 0.039 | | 2,630 | | 0.051 |
| Others | 1,806 | 0.015 | | 2,121 | | 0.007 | 2,443 | | 0.025 | | 2,630 | | 0.004 |
| Table S3. Descriptive statistics for Liberia | | | | | | | | | | | | | |
|  | | | | | | | | | | | | | |
|  | **Pre-Ebola** | | | | **Pre-Ebola** | | | | | **Post-Ebola** | | | |
|  | 2007 | | | | 2013 | | | | | 2019 | | | |
|  | *Observations* | | *Mean* | | *Observations* | | | *Observations* | | *Mean* | | *Observations* | |
|  |  | |  | |  | | |  | |  | |  | |
| Fully immunized | 1,987 | | 0.243 | | 2,641 | | | 0.412 | | 1,914 | | 0.427 | |
| Age | 1,987 | | 1.998 | | 2,641 | | | 1.940 | | 1,914 | | 1.973 | |
| Girl | 1,987 | | 0.500 | | 2,641 | | | 0.485 | | 1,914 | | 0.527 | |
| Birth order of the child | 1,987 | | 3.600 | | 2,641 | | | 3.743 | | 1,914 | | 3.664 | |
| Mother’s age | 1,987 | | 28.500 | | 2,641 | | | 28.203 | | 1,914 | | 28.776 | |
| Mother’s education | 1,986 | | 0.713 | | 2,641 | | | 0.753 | | 1,914 | | 0.853 | |
| Christian | 1,987 | | 0.862 | | 2,641 | | | 0.842 | | 1,914 | | 0.837 | |
| Improved toilet | 1,945 | | 0.790 | | 2,581 | | | 0.707 | | 1,866 | | 0.689 | |
| Earth floor | 1,954 | | 0.656 | | 2,586 | | | 0.693 | | 1,871 | | 0.603 | |
|  |  | |  | |  | | |  | |  | |  | |
| *Wealth quantile* |  | |  | |  | | |  | |  | |  | |
| Quantile 1 (poorest) | 1,987 | | 0.253 | | 2,641 | | | 0.363 | | 1,914 | | 0.354 | |
| Quantile 2 | 1,987 | | 0.230 | | 2,641 | | | 0.283 | | 1,914 | | 0.285 | |
| Quantile 3 | 1,987 | | 0.232 | | 2,641 | | | 0.200 | | 1,914 | | 0.196 | |
| Quantile 4 | 1,987 | | 0.188 | | 2,641 | | | 0.103 | | 1,914 | | 0.099 | |
| Quantile 5 (richest) | 1,987 | | 0.097 | | 2,641 | | | 0.051 | | 1,914 | | 0.066 | |
|  |  | |  | |  | | |  | |  | |  | |
| Female household head | 1,987 | | 0.303 | | 2,641 | | | 0.307 | | 1,914 | | 0.325 | |
| Age of household head | 1,987 | | 40.704 | | 2,641 | | | 40.039 | | 1,914 | | 40.900 | |
| Urban | 1,987 | | 0.342 | | 2,641 | | | 0.303 | | 1,914 | | 0.297 | |
| Population density | 1,987 | | -3.985 | | 2,641 | | | -4.732 | | 1,914 | | -4.544 | |
| Rainfall | 1,987 | | 2671.0 | | 2,641 | | | 3291.0 | | 1,914 | | 2227.5 | |

| Table S4. Descriptive statistics for Sierra Leone | | | | | | |
| --- | --- | --- | --- | --- | --- | --- |
|  | | | | | | |
|  | **Pre-Ebola** | | **Pre-Ebola** | | **Post-Ebola** | |
|  | 2008 | | 2013 | | 2019 | |
|  | *Observations* | *Mean* | *Observations* | *Observations* | *Mean* | *Observations* |
|  |  |  |  |  |  |  |
| Fully immunized | 1,868 | 0.375 | 4,013 | 0.702 | 3,374 | 0.531 |
| Age | 1,868 | 1.845 | 4,013 | 1.890 | 3,374 | 1.929 |
| Girl | 1,868 | 0.495 | 4,013 | 0.511 | 3,374 | 0.502 |
| Birth order of the child | 1,868 | 3.405 | 4,013 | 3.582 | 3,374 | 3.328 |
| Mother’s age | 1,868 | 28.374 | 4,013 | 28.537 | 3,374 | 28.707 |
| Mother’s education | 1,868 | 0.383 | 4,013 | 0.517 | 3,374 | 0.760 |
| Muslim | 1,868 | 0.225 | 4,013 | 0.185 | 3,374 | 0.198 |
| Improved toilet | 1,848 | 0.588 | 3,985 | 0.537 | 3,345 | 0.541 |
| Earth floor | 1,811 | 0.653 | 3,975 | 0.638 | 3,344 | 0.528 |
|  |  |  |  |  |  |  |
| *Wealth quantile* |  |  |  |  |  |  |
| Quantile 1 (poorest) | 1,868 | 0.222 | 4,013 | 0.241 | 3,374 | 0.258 |
| Quantile 2 | 1,868 | 0.200 | 4,013 | 0.207 | 3,374 | 0.236 |
| Quantile 3 | 1,868 | 0.207 | 4,013 | 0.201 | 3,374 | 0.219 |
| Quantile 4 | 1,868 | 0.212 | 4,013 | 0.218 | 3,374 | 0.172 |
| Quantile 5 (richest) | 1,868 | 0.159 | 4,013 | 0.134 | 3,374 | 0.115 |
|  |  |  |  |  |  |  |
| Female household head | 1,868 | 0.186 | 4,013 | 0.256 | 3,374 | 0.226 |
| Age of household head | 1,868 | 43.104 | 4,013 | 43.379 | 3,374 | 42.931 |
| Urban | 1,868 | 0.310 | 4,013 | 0.284 | 3,374 | 0.293 |
| Population density | 1,868 | -4.718 | 4,013 | -5.029 | 3,374 | -4.238 |
| Rainfall | 1,868 | 2464.5 | 4,013 | 2857.7 | 3,374 | 2521.0 |
|  |  |  |  |  |  |  |
|  |  |  |  |  |  |  |
|  |  |  |  |  |  |  |
|  |  |  |  |  |  |  |
|  |  |  |  |  |  |  |
|  |  |  |  |  |  |  |
|  |  |  |  |  |  |  |
|  |  |  |  |  |  |  |
|  |  |  |  |  |  |  |
| Table S4 Descriptive statistics for Sierra Leone (continued) | | | | | | |
|  | | | | | | |
|  | **Pre-Ebola** | | **Pre-Ebola** | | **Post-Ebola** | |
|  | 2008 | 2013 | 2019 | 2008 | 2013 | 2019 |
|  | *Observations* | *Mean* | *Observations* | *Observations* | *Mean* | *Observations* |
|  |  |  |  |  |  |  |
| *Ethnicity* |  |  |  |  |  |  |
| Creole | 1,868 | 0.008 | 4,013 | 0.005 | 3,374 | 0.004 |
| Fullah | 1,868 | 0.000 | 4,013 | 0.029 | 3,374 | 0.036 |
| Kono | 1,868 | 0.074 | 4,013 | 0.055 | 3,374 | 0.037 |
| Limba | 1,868 | 0.058 | 4,013 | 0.058 | 3,374 | 0.077 |
| Loko | 1,868 | 0.017 | 4,013 | 0.022 | 3,374 | 0.017 |
| Mandingo | 1,868 | 0.022 | 4,013 | 0.023 | 3,374 | 0.020 |
| Mende | 1,868 | 0.397 | 4,013 | 0.365 | 3,374 | 0.365 |
| Sherbro | 1,868 | 0.015 | 4,013 | 0.026 | 3,374 | 0.026 |
| Temne | 1,868 | 0.278 | 4,013 | 0.314 | 3,374 | 0.309 |
| Korankoh | 1,868 | 0.000 | 4,013 | 0.044 | 3,374 | 0.055 |
| Others | 1,868 | 0.132 | 4,013 | 0.058 | 3,374 | 0.056 |

| Table S5. The number of observations in each prefecture or district and in each round | | | | | | | | | |
| --- | --- | --- | --- | --- | --- | --- | --- | --- | --- |
|  |  |  |  |  |  |  |  |  |  |
| Guinea prefecture | 1999 | 2005 | 2012 | 2018 |  | Liberia district | 2007 | 2013 | 2019 |
| Beyla | 48 | 53 | 66 | 71 |  | Bomi | 35 | 111 | 80 |
| Boffa | 33 | 47 | 55 | 95 |  | Bong | 97 | 182 | 166 |
| Boke | 70 | 78 | 100 | 129 |  | Gbarpolu | 134 | 152 | 102 |
| Conakry | 179 | 214 | 234 | 258 |  | Grand Bassa | 65 | 137 | 138 |
| Coyah | 24 | 8 | 33 | 33 |  | Grand Cape Mount | 55 | 186 | 110 |
| Dabola | 26 | 62 | 68 | 63 |  | Grand Gedeh | 133 | 131 | 82 |
| Dalaba | 30 | 42 | 53 | 48 |  | Grand Kru | 36 | 142 | 112 |
| Dinguiraye | 36 | 61 | 84 | 88 |  | Lofa | 74 | 171 | 125 |
| Dubreka | 43 | 24 | 79 | 58 |  | Margibi | 146 | 171 | 96 |
| Faranah | 43 | 50 | 87 | 86 |  | Maryand | 196 | 163 | 115 |
| Forecariah | 31 | 45 | 35 | 44 |  | Montserrado | 415 | 225 | 139 |
| Fria | 17 | 28 | 27 | 36 |  | Nimba | 139 | 285 | 212 |
| Gaoual | 34 | 58 | 30 | 57 |  | River Gee | 67 | 144 | 87 |
| Gueckedou | 100 | 56 | 70 | 66 |  | Rivercess | 31 | 158 | 93 |
| Kankan | 80 | 84 | 129 | 93 |  | Sinoe | 85 | 163 | 93 |
| Kerouane | 40 | 64 | 57 | 47 |  |  |  |  |  |
| Kindia | 102 | 104 | 100 | 98 |  | Sierra Leone district | 2008 | 2013 | 2019 |
| Kissidougou | 55 | 90 | 118 | 85 |  | Bo | 184 | 337 | 260 |
| Koubia | 18 | 22 | 30 | 40 |  | Bombali | 106 | 245 | 211 |
| Koundara | 30 | 25 | 23 | 44 |  | Bonthe | 62 | 213 | 209 |
| Kouroussa | 52 | 31 | 38 | 54 |  | Falaba | 0 | 0 | 136 |
| Labe | 52 | 75 | 104 | 89 |  | Kailahun | 116 | 304 | 175 |
| Lelouma | 32 | 32 | 55 | 48 |  | Kambia | 120 | 328 | 237 |
| Lola | 21 | 31 | 35 | 27 |  | Karene | 0 | 0 | 174 |
| Macenta | 104 | 81 | 30 | 53 |  | Kenema | 195 | 285 | 282 |
| Mali | 36 | 35 | 67 | 90 |  | Koinadugu | 107 | 310 | 168 |
| Mamou | 38 | 113 | 111 | 95 |  | Kono | 207 | 318 | 185 |
| Mandiana | 41 | 29 | 67 | 48 |  | Moyamba | 96 | 272 | 223 |
| Nzerekore | 113 | 44 | 96 | 95 |  | Port Loko | 115 | 355 | 210 |
| Pita | 25 | 77 | 85 | 82 |  | Pujehun | 88 | 263 | 194 |
| Siguiri | 48 | 56 | 104 | 173 |  | Tonkolili | 156 | 301 | 214 |
| Telimele | 53 | 45 | 57 | 75 |  | Western Rural | 73 | 201 | 199 |
| Tougue | 14 | 37 | 41 | 49 |  | Western Urban | 127 | 161 | 139 |
| Yomou | 24 | 22 | 9 | 15 |  |  |  |  |  |

| Table S6. Estimated ranges of Ebola incidence in each prefecture / district | | | | |
| --- | --- | --- | --- | --- |
|  |  |  |  |  |
| Guinea prefecture | incidence per 100K |  | Liberia district | incidence per 100K |
| Beyla | 10.1−25.0 |  | Bomi | > 100 |
| Boffa | 10.1−25.0 |  | Bong | 25.1−50.0 |
| Boke | 0.1−10.0 |  | Gbarpolu | 10.1−25.0 |
| Conakry | > 100 |  | Grand Bassa | 10.1−25.0 |
| Coyah | 50.1−100.0 |  | Grand Cape Mount | 50.1−100.0 |
| Dabola | 0.1−10.0 |  | Grand Gedeh | 0.1−10.0 |
| Dalaba | 0.1−10.0 |  | Grand Kru | 0.1−10.0 |
| Dinguiraye | 0.0 |  | Lofa | > 100 |
| Dubreka | 25.1−50.0 |  | Margibi | > 100 |
| Faranah | 10.1−25.0 |  | Maryand | 0.1−10.0 |
| Forecariah | > 100 |  | Montserrado | > 100 |
| Fria | 10.1−25.0 |  | Nimba | 25.1−50.0 |
| Gaoual | 0.0 |  | River Gee | 10.1−25.0 |
| Gueckedou | 50.1−100.0 |  | Rivercess | 25.1−50.0 |
| Kankan | 0.1−10.0 |  | Sinoe | 10.1−25.0 |
| Kerouane | 50.1−100.0 |  |  |  |
| Kindia | 10.1−25.0 |  | Sierra Leone district | incidence |
| Kissidougou | 25.1−50.0 |  | Bo | 50.1−100.0 |
| Koubia | 0.0 |  | Bombali | > 100 |
| Koundara | 0.0 |  | Bonthe | 0.1−10.0 |
| Kouroussa | 0.1−10.0 |  | Falaba | 25.1−50.0 |
| Labe | 0.0 |  | Kailahun | > 100 |
| Lelouma | 0.0 |  | Kambia | 50.1−100.0 |
| Lola | 50.1−100.0 |  | Karene | > 100 |
| Macenta | > 100 |  | Kenema | > 100 |
| Mali | 0.1−10.0 |  | Koinadugu | 25.1−50.0 |
| Mamou | 0.0 |  | Kono | 50.1−100.0 |
| Mandiana | 0.0 |  | Moyamba | 50.1−100.0 |
| Nzerekore | 50.1−100.0 |  | Port Loko | > 100 |
| Pita | 0.1−10.0 |  | Pujehun | 10.1−25.0 |
| Siguiri | 0.1−10.0 |  | Tonkolili | > 100 |
| Telimele | 10.1−25.0 |  | Western Rural | > 100 |
| Tougue | 0.1−10.0 |  | Western Urban | > 100 |
| Yomou | 0.1−10.0 |  |  |  |

| Table S7. Vaccination rates in high-incidence and low-incidence prefectures / districts | | | | |
| --- | --- | --- | --- | --- |
|  |  |  |  |  |
|  | threshold = 10 per 100K | | threshold = 25 per 100K | |
| Guinea | *high-incidence* | *low-incidence* | *high-incidence* | *low-incidence* |
| Fraction of children fully immunized | 0.30 | 0.26 | 0.33 | 0.26 |
| Mean vaccinations per child / 9 | 0.65 | 0.57 | 0.68 | 0.57 |
|  |  |  |  |  |
|  | threshold = 25 per 100K | | threshold = 50 per 100K | |
| Liberia | *high-incidence* | *low-incidence* | *high-incidence* | *low-incidence* |
| Fraction of children fully immunized | 0.43 | 0.30 | 0.46 | 0.33 |
| Mean vaccinations per child / 9 | 0.77 | 0.68 | 0.79 | 0.71 |
|  |  |  |  |  |
|  | threshold = 100 per 100K | |  | |
| Sierra Leone | *high-incidence* | *low-incidence* |  |  |
| Fraction of children fully immunized | 0.57 | 0.59 |  |  |
| Mean vaccinations per child / 9 | 0.83 | 0.83 |  |  |

| Table S8. Estimates of the effect on vaccination status of residing in an Ebola-affected district | | | | | | | |
| --- | --- | --- | --- | --- | --- | --- | --- |
|  | | | | | | | |
|  | | Probit model of whether a child is fully vaccinated | | | Fractional Probit model of the fraction of vaccinations received | | |
| *country* | *cut-off* | *coeff. (**)* | *std. err.* | *a.m.e.* | *coeff. (*$\hat{\eta}_{3}$*)* | *std. err.* | *a.m.e.* |
| **Guinea** | **10 per 100K** | **–0.553**** | **0.219** | **–0.144** | **–0.237**** | **0.209** | **–0.084** |
| **Guinea** | **25 per 100K** | **–0.576**** | **0.227** | **–0.151** | **–0.427**** | **0.165** | **–0.153** |
| Guinea | 50 per 100K | *–0.649*** | *0.268* | *–0.169* | –0.343** | 0.200 | –0.123 |
| Guinea | 100 per 100K | –0.882** | 0.216 | –0.229 | –0.404** | 0.153 | –0.145 |
| Liberia | 10 per 100K | *0.249*** | *0.104* | *0.093* | *0.231*** | *0.090* | *0.064* |
| **Liberia** | **25 per 100K** | **–0.353**** | **0.224** | **–0.132** | **–0.429**** | **0.115** | **–0.120** |
| **Liberia** | **50 per 100K** | **–0.145**** | **0.232** | **–0.054** | **–0.256**** | **0.162** | **–0.071** |
| Liberia | 100 per 100K | –0.141** | 0.230 | –0.053 | –0.231** | 0.168 | –0.064 |
| Sierra Leone | 10 per 100K | 0.104** | 0.109 | 0.042 | 0.167** | 0.061 | 0.039 |
| Sierra Leone | 25 per 100K | –0.056** | 0.148 | –0.021 | 0.032** | 0.102 | 0.007 |
| Sierra Leone | 50 per 100K | –0.120** | 0.097 | –0.046 | –0.075** | 0.098 | –0.017 |
| **Sierra Leone** | **100 per 100K** | ***–0.285***** | ***0.104*** | ***–0.110*** | **–0.405**** | **0.081** | **–0.094** |
| *Cut-off* indicates the threshold incidence used to define whether a child’s district has been affected by Ebola. *Coeff.* indicates a regression coefficient, *std. err.* indicates a standard error, and *a.m.e.* indicates an average marginal effect. ** indicates a coefficient significantly different from zero at the one percent level, and * indicates significance at the five percent level. Results in bold are reported in the main text: these are cases in which the proportion of children in Ebola-affected districts is over 30% and under 70%. Results in italics are cases in which the parallel trends assumption can be rejected at the 5 percent level using the method in section S2. | | | | | | | |

Figure S1. Tests of the parallel trends assumption in the Guinean models.

For each model, for each definition of high Ebola incidence, and for each period (*t* = 1,2,3) the dots show the estimated average effect of living in a high-incidence district in period *t*; the bars show the corresponding Bonferroni-corrected 95 percent confidence intervals. Here, period 1 = the 2005 survey, period 2 = the 2012 survey, and period 3 = the 2018 survey. In the Probit model, the outcome is the probability of being fully vaccinated; in the fractional model, the outcome is the fraction of vaccinations received. The treatment is in period 3: under the null, the effects in periods 1-2 are equal to zero.

Figure S2. Tests of the parallel trends assumption in the Liberian models.

For each model, for each definition of high Ebola incidence, and for each period (*t* = 1,2) the dots show the estimated average effect of living in a high-incidence district in period *t*; the bars show the corresponding Bonferroni-corrected 95 percent confidence intervals. Here, period 1 = the 2013 survey and period 2 = the 2019 survey. In the Probit model, the outcome is the probability of being fully vaccinated; in the fractional model, the outcome is the fraction of vaccinations received. The treatment is in period 2: under the null, the effect in period 1 is equal to zero.

Figure S3. Tests of the parallel trends assumption in the Sierra Leone models.

For each model and for each period (*t* = 1,2) the dots show the estimated average effect of living in a high-incidence district in period *t*; the bars show the corresponding Bonferroni-corrected 95 percent confidence intervals. Here, period 1 = the 2013 survey and period 2 = the 2019 survey. In the Probit model, the outcome is the probability of being fully vaccinated; in the fractional model, the outcome is the fraction of vaccinations received. The treatment is in period 2: under the null, the effect in period 1 is equal to zero.
